# Experimental Assessment of Trigger-Based MU-OFDMA for Deterministic Wi-Fi 6 Operation on COTS Devices

**DOI:** 10.3390/s26113416

**Published:** 2026-05-28

**Authors:** Federico Orozco-Santos, Víctor Sempere-Payá, Javier Silvestre-Blanes

**Affiliations:** 1 Instituto Tecnológico de Informática (ITI), 46980 Valencia, Spain; 2 Departamento de Comunicaciones (DCOM), Universitat Politècnica de València (UPV), 46022 Valencia, Spain; vsempere@dcom.upv.es; 3 Departamento de Informática de Sistemas y Computadores (DISCA), Universitat Politècnica de València (UPV), 03801 Alcoy, Spain; jsilves@disca.upv.es

**Keywords:** determinism, OFDMA, QoS, TUA, TWT, Wi-Fi, wireless

## Abstract

Wireless networks are increasingly considered for industrial and time-critical applications, where flexible deployment must be reconciled with predictable communication behaviour. IEEE 802.11ax introduces mechanisms such as Orthogonal Frequency Division Multiple Access (OFDMA), Trigger-based Uplink Access (TUA), and Target Wake Time (TWT) as part of ongoing efforts to support bounded latency and deterministic transmissions in Wi-Fi networks. However, the practical behaviour of these mechanisms depends not only on the standard, but also on what commercial devices expose, how access points implement scheduling decisions, and how trigger-based access, RU assignment, and timing control can be configured in real deployments. This paper therefore focuses on the practical implementation and experimental assessment of OFDMA-based deterministic operation using Wi-Fi 6 commercial off-the-shelf (COTS) hardware. The proposed configuration combines driver-level enabling of high-efficiency mechanisms with controlled testbed measurements and complementary simulations, allowing OFDMA operation to be compared against conventional single-user OFDM under realistic traffic and interference conditions. The results show that coordinated OFDMA operation on COTS devices improves temporal stability, reducing jitter by up to 23% and latency by approximately 44% with respect to single-user OFDM operation. The experiments also reveal practical effects that are central to deterministic-oriented Wi-Fi: simultaneous RU-based transmissions reduce contention-driven variability, TWT-based activity windows improve temporal alignment, and RU subdivision introduces a throughput trade-off that must be considered when dimensioning industrial traffic. Overall, the study provides empirical evidence that Wi-Fi 6 can support deterministic-oriented industrial communication when OFDMA, trigger-based access, and timing mechanisms are jointly configured, while also highlighting the implementation constraints that remain when moving from standard capabilities to COTS device behaviour.

## 1. Introduction

Wireless networks have become a cornerstone of modern connectivity, with their deployment rapidly expanding across a wide range of scenarios. This growth has increased network density and increased interference issues. To address these challenges, the IEEE 802 LAN/MAN standardisation committee introduced the IEEE 802.11ax amendment [1], which differs from its predecessors (e.g., 802.11ac and 802.11n) by prioritising user-layer efficiency rather than merely increasing nominal data rates.

IEEE 802.11ax introduces several mechanisms to improve user-layer efficiency, including higher modulation and coding schemes (MCSs), overlapping basic service set (BSS) management, spatial reuse, advanced power management, uplink multi-user multiple-input multiple-output (MU-MIMO), and orthogonal frequency division multiple access (OFDMA). The main objective of OFDMA is to partition the available channel bandwidth amongst multiple users, enabling multiple stations (STAs) to transmit and/or receive data concurrently. Compared with conventional non-OFDMA transmissions, this approach provides a more flexible and efficient use of channel resources.

In uplink scenarios, OFDMA offers additional benefits by concentrating each STA’s transmit power into narrow frequency subbands, thereby facilitating the use of higher MCSs [2]. However, OFDMA performance depends strongly on how channel resources are allocated amongst STAs, which is determined by the resource-scheduling mechanism. Legacy methods, in which the entire channel is assigned to a single STA at a time, are often inefficient when many STAs transmit small packets, as a substantial fraction of channel time is consumed by protocol overhead [3]. Therefore, advanced schedulers that exploit simultaneous transmissions across multiple STAs are critical to maximising OFDMA’s potential [4]. Although this study focuses primarily on uplink OFDMA, several findings can be generalised to downlink scenarios, where resource allocation is typically less complex.

OFDMA schedulers have been widely studied in the context of general wireless communication systems, where optimisation problems are commonly formulated to allocate tones and transmit power to users, with the aim of maximising throughput under power constraints. However, many studies assume ideal conditions, such as perfect channel state information (CSI), unrestricted tone assignments, and arbitrary power distribution, which simplify the optimisation process [5]. These assumptions enable exact solutions and linear computation times with respect to the number of users, tones, and MCSs. In practice, real wireless systems, including IEEE 802.11ax, impose additional constraints that require new approaches. In particular, IEEE 802.11ax introduces constraints such as fixed resource unit (RU) positions and sizes and the requirement to equalise transmission power levels across STAs. Furthermore, frequency-selective fading, where different parts of the band experience different attenuation levels, must be considered during scheduling. Existing work on OFDMA scheduling for IEEE 802.11ax often overlooks these practical challenges by assuming ideal CSI and neglecting the impact of fading on RU allocation. Moreover, in real deployments, perfect CSI is rarely available, which further complicates resource-allocation decisions.

These recent enhancements in IEEE 802.11ax have shifted the focus from a purely incremental improvement in physical-layer performance towards a far more coordinated access architecture, in which OFDMA introduces spectral granularity through RUs, uses trigger frames to coordinate multi-user transmissions, and is complemented by additional timing-control mechanisms that open the door to traffic with stricter latency and predictability requirements [6]. Within this context, a first strand of the literature has examined the fundamental behaviour of OFDMA in Wi-Fi 6 networks, assessing its impact on throughput, delay, and access efficiency as a function of load, traffic mix, and interaction with legacy stations. Along these lines, simulation-based results have shown that OFDMA can significantly reduce median latency and increase aggregate throughput when it is properly configured together with buffer-status reporting and access-control mechanisms [7]. Complementarily, joint analytical and simulation studies have shown that the actual gains of 802.11ax depend critically on the scheduling logic implemented at the AP, particularly in the presence of differentiated channel access, mixed traffic, and coexistence with legacy nodes [8]. These findings are consistent with dedicated scheduling proposals such as the maximum-throughput scheduler presented in [9], which formulates RU allocation by considering load, MCS, and traffic ageing. Taken together, this body of work shows that OFDMA should not be viewed merely as a physical-layer orthogonal multiplexing mechanism, but rather as an access framework whose effective performance emerges from the interaction between RU selection, uplink and downlink coordination, aggregation policies, and access timing. This perspective naturally leads to the notion of quality of service (QoS), understood here as the ability of the network to differentiate between traffic classes and to meet distinct requirements in throughput, delay, and reliability.

Building on this foundation, a second line of work has addressed dynamic allocation and QoS-aware scheduling, explicitly considering scenarios in which users with heterogeneous requirements coexist. In this direction, Ref.  [10] propose an algorithm for OFDMA resource allocation in the simultaneous presence of latency-sensitive and high-throughput stations, showing that scheduling policy directly shapes the trade-off between delay and capacity. This discussion is extended in [3] to uplink random access, where a quasi-scheduled access scheme and state-dependent resource allocation are introduced to improve responsiveness in industrial IoT environments. Likewise, Ref. [11] broaden the analysis to the Age of Information metric, demonstrating that the optimisation of uplink random access parameters substantially affects information freshness in 802.11ax networks. Collectively, these studies reinforce an important conclusion: OFDMA provides a highly flexible framework for decoupling users, flows, and priorities, but deterministic or quasi-deterministic performance depends on how its access mechanisms are combined with higher-layer MAC decisions. Nevertheless, this works still relies on simulation or analytical modelling, leaving open the relationship between the reported behaviour and the constraints imposed by implementation on commercial chipsets.

A third line of research, closer to industrial and real-time applications, has begun to examine OFDMA not only as an efficiency mechanism, but also as a means of temporal control, wireless TSN integration, and prioritised access provisioning. In this context, Ref. [6] study how Wi-Fi 6 can be used in industrial scenarios, summarising the main lessons learned from factory-automation use cases and showing that, with a suitable configuration of its available features, Wi-Fi 6 can meet demanding QoS requirements for IIoT applications. In a related direction, Refs. [4,12] analyse the integration of UL-OFDMA with time-sensitive wireless networks, showing that the explicit exposure of timing requirements at the endpoints and QoS-oriented resource allocation can drastically reduce latency and improve channel utilisation. Similarly, Ref. [13] combine OFDMA with TSN-inspired mechanisms such as priority scheduling and traffic shaping for healthcare applications with stringent delay constraints. Moreover, Ref. [2] specifically study the use of triggering in 802.11ax within a TCP-based networked control application, showing that merely enabling OFDMA does not guarantee latency improvements and may, in some contexts, even degrade temporal behaviour if it is not harmonised with traffic dynamics and access policy. Finally, Refs. [14,15] extend the discussion to Wi-Fi 7 and beyond, where the relevant novelty is not only an abstract improvement in efficiency but the introduction of concrete control mechanisms that generalise the behaviour evaluated in this paper. Restricted TWT (rTWT) extends the TWT-based activity windows considered here by reserving protected service periods for latency-sensitive traffic, which is directly connected to our observation that temporal stability emerges only when wake/sleep timing and trigger-based uplink opportunities are aligned. Multi-RU allocation extends the RU assignment evaluated in our 20 MHz IEEE 802.11ax testbed by allowing a station or flow to be mapped onto more flexible frequency-domain resources; therefore, the throughput–latency trade-off and the RU-level isolation measured in this work become explicit scheduling variables in Wi-Fi 7. Multi-Link Operation (MLO) further moves the same problem from a single channel to several coordinated links, so the station-level isolation observed with MU-OFDMA can be extended through link selection, traffic steering, or duplication of critical flows. Looking towards Wi-Fi 8, Multi-AP Coordination mechanisms, including Coordinated OFDMA, Coordinated Spatial Reuse, and Coordinated Beamforming, extend the same principles to neighbouring APs: trigger timing, RU assignment, transmit power, and interference directionality must be jointly controlled across BSSs. Overall, these studies show that the key challenge is no longer simply to use OFDMA, but rather to coordinate OFDMA, trigger-based access, TWT-like reservation, link selection, and inter-AP coordination in order to build controllable temporal behaviour in shared wireless networks.

Despite these advances, a structural limitation remains in the state of the art. On the one hand, analytical and simulation-based studies make it possible to explore scalability, parameter sensitivity, and behaviour under idealised conditions, but they do not fully capture the constraints imposed by firmware, drivers, configuration granularity, internal AP timing, or the proprietary implementation of scheduling logic. On the other hand, many of the most advanced experimental studies rely on SDR platforms, which provide a degree of instrumentation and control that is simply unavailable in closed commercial devices [2,14]. As a result, a clear separation still exists between what the 802.11ax standard enables in theory, what simulators and open prototypes can demonstrate, and what COTS devices actually implement and expose in real operating scenarios, a distinction that becomes especially relevant when OFDMA, TWT, and trigger frames are combined, because the interaction between these mechanisms depends on internal vendor decisions, MAC timings that are not always visible, and practical limitations that are rarely reflected in simulation.

This gap provides the direct technical motivation for the present work, whose purpose is to experimentally validate the joint implementation of OFDMA, TWT, and trigger-based mechanisms on commercial off-the-shelf (COTS) devices, thereby complementing the existing literature on modelling, simulation, and SDR-based platforms with empirical evidence on the practical feasibility of deploying these mechanisms for applications with strict requirements in latency, efficiency, and temporal predictability. More specifically, this paper presents a comprehensive experimental evaluation of OFDMA’s capability to support deterministic behaviour in Wi-Fi 6 networks, focusing on the extent to which the advanced capabilities defined in IEEE 802.11ax become observable, configurable, and reproducible on commercial hardware, how they interact when activated simultaneously, and what effects they have on temporal behaviour and link efficiency under realistic traffic conditions. Unlike prior work, which often relies on theoretical models or custom hardware, this study does not treat latency and jitter reductions as the only novelty. Instead, it examines the implementation-level effects that explain when those reductions become reproducible on COTS devices and when they do not follow directly from the theoretical availability of OFDMA. The key contributions of this work are as follows:

- Implementation dependent determinism on COTS hardware: Deterministic behaviour is found to depend on the joint configuration of TWT scheduling, Trigger-based Uplink Access, and fixed RU assignment, rather than on OFDMA activation alone.
- Gap between standard-level capabilities and commercial implementation: The evaluation exposes the mismatch between the scheduling flexibility defined by IEEE 802.11ax and the subset of OFDMA control variables that can be accessed and enforced through commercial chipset firmware and driver interfaces.
- Load and interference-resilient variance containment: The experiments reveal that scheduled RU-based access improves determinism mainly by containing jitter and tail-latency growth under increasing load and external interference, rather than only by reducing mean latency. This effect is reinforced by narrow RU operation, where transmit power is concentrated over reduced bandwidths, linking OFDMA scheduling with physical-layer robustness on COTS hardware.

Beyond corroborating throughput and delay trends that have already been reported in the literature using numerical studies, our work highlights a less explored and non-obvious dimension of IEEE 802.11ax determinism: the extent to which TWT and TUA can be orchestrated on COTS devices to obtain repeatable uplink timing behaviour. In this paper, the corresponding configuration choices are performed manually in order to isolate the effect of each mechanism and to provide a reproducible baseline. This exposes a relevant device-level distinction: the standard provides the primitives for scheduled uplink access, but the observable deterministic behaviour depends on the granularity and timing discipline that the AP firmware and driver allow the experimenter to control. Therefore, the main experimental insight is not simply that OFDMA reduces delay, but that deterministic-oriented Wi-Fi 6 requires aligning three control planes that are usually hidden or partially automated in commercial equipment: sleep/wake negotiation through TWT, trigger generation by the AP, and RU-level frequency allocation. By exposing and controlling parameters that are typically selected internally by vendor firmware, we show that medium access can be steered towards deterministic operation and, importantly, that the associated control plane can in principle be externalised.

This work offers a focused contribution by demonstrating the feasibility of deterministic-oriented medium access in 802.11ax networks using existing OFDMA and TUA functionalities on COTS devices, thereby providing an experimental baseline for future evaluations in larger and more heterogeneous time-sensitive Wi-Fi scenarios.

The remainder of this paper is organised as follows. Section 2 provides the technical background on OFDMA, including RU allocation and the TUA and TWT mechanisms. Section 3 presents the interference models considered in the study. Section 4 and Section 5 report the experimental methodology and performance results for single-client and multi-client scenarios, respectively. Finally, Section 6 concludes the paper and outlines future research directions.

## 2. Overview

Orthogonal Frequency Division Multiple Access (OFDMA) is a frequency-domain multiple-access mechanism built on top of OFDM, the multicarrier physical-layer waveform used in IEEE 802.11 systems and in other wireless technologies such as LTE and 5G NR [16,17]. In IEEE 802.11ax, OFDMA enables a Wi-Fi channel to be partitioned into smaller orthogonal frequency-domain allocations known as Resource Units (RUs) [16,18]. By assigning different RUs to different stations (STAs), an Access Point (AP) can coordinate simultaneous transmissions from or to multiple users within the same transmission opportunity (TXOP), depending on the downlink or uplink scheduling configuration [18,19].

This frequency-domain multiplexing should be distinguished from conventional non-OFDMA channel access. In non-OFDMA operation, a transmission occupies the full channel bandwidth from the frequency-domain resource-allocation perspective, rather than dividing the channel into separate RUs assigned to different users. This does not imply that previous Wi-Fi generations were strictly single-user in all cases: for example, IEEE 802.11ac introduced downlink multi-user MIMO (DL MU-MIMO), which allows simultaneous downlink transmission to multiple users through spatial multiplexing. However, DL MU-MIMO and OFDMA exploit different resource dimensions. MU-MIMO separates users in the spatial domain, whereas OFDMA separates users in the frequency domain by assigning different RUs. The distinction considered in this work is therefore the use of OFDMA-based RU partitioning and scheduling, rather than a change in the underlying OFDM waveform itself.

### 2.1. OFDMA Transmission and Scheduling

**Figure 1:**
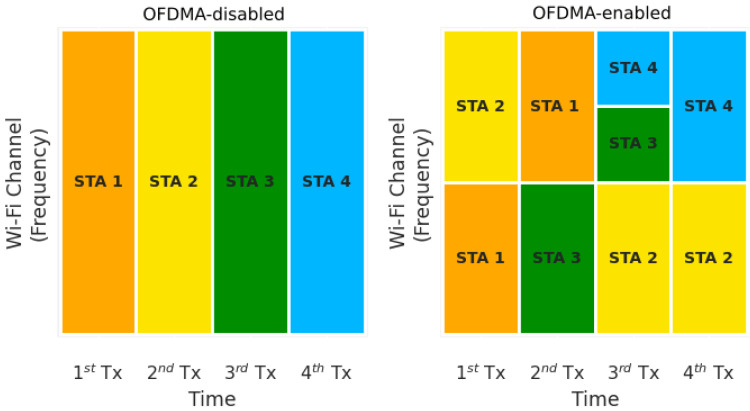
Conceptual comparison between OFDMA-disabled and OFDMA-enabled channel access in IEEE 802.11ax.

The operational distinction between OFDMA-disabled and OFDMA-enabled channel access is visually represented in Figure 1. When OFDMA is disabled, the system operates in a conventional single-user (SU) transmission mode, as depicted on the left, where the entire Wi-Fi channel bandwidth is allocated to one station at a time. When OFDMA is enabled, the available time-frequency resources are partitioned into Resource Units (RUs), which can be assigned to multiple STAs within the same transmission opportunity, as depicted on the right. In both cases, OFDM remains the underlying physical-layer multicarrier modulation; the difference lies in whether the channel resources are assigned to a single user or shared among multiple users through OFDMA. This parallel communication significantly improves the overall network efficiency, particularly in multi-user environments [20,21,22].

Resource Units (RUs) are the fundamental building blocks of OFDMA, comprising groups of subcarriers, also known as tones, that are allocated to specific users [16,23,24]. The IEEE 802.11ax standard defines various RU sizes, including 26, 52, 106, 242, 484, and 996 tones [24,25]. The smallest RU type consists of 26 subcarriers, occupying a minimum bandwidth of 2 MHz [25]. A crucial aspect of OFDMA is the reduced subcarrier spacing in Wi-Fi 6. While OFDM typically uses 64 subcarriers spaced 312.5 kHz apart within a 20 MHz channel, OFDMA in 802.11ax employs 256 subcarriers spaced 78.125 kHz apart, representing a fourfold increase in subcarrier count and a fourfold reduction in spacing [26]. This narrower subcarrier spacing is accompanied by a fourfold increase in the OFDM symbol duration, from 3.2 µs in 802.11ac to 12.8 µs in 802.11ax (excluding guard intervals) [26]. These changes collectively enhance the robustness in multipath fading environments and facilitate the multi-user capabilities of OFDMA. The allocation of these RUs is centrally controlled by the AP, which dynamically assigns them based on the data transmission requirements of each device [18,19].

**Figure 2:**
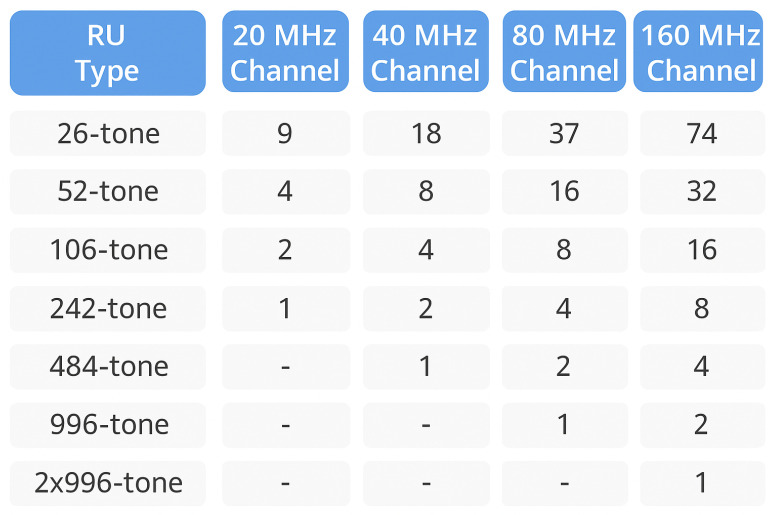
Number of STAs as a function of the resource unit size and channel bandwidth.

The relationship between RU type and channel bandwidth is summarised in Figure 2. As shown, the number of available RUs increases proportionally with the channel bandwidth, enabling finer-grained multi-user scheduling. For example, a 20 MHz channel can be subdivided into up to nine 26-tone RUs, while a 160 MHz channel supports up to seventy-four 26-tone RUs. Larger RU types, such as 242-tone or 996-tone, occupy broader frequency segments and thus allow fewer simultaneous allocations within the same channel. This hierarchical RU structure provides flexibility for access points to balance throughput and latency: smaller RUs support multiple low-rate or latency-sensitive transmissions, whereas larger RUs are better suited for high-throughput users. Consequently, the RU configuration plays a central role in achieving deterministic and efficient medium access in Wi-Fi 6 OFDMA systems.

To make this configurability explicit for the 20 MHz case, Table 1 reproduces the official RU allocation patterns defined by IEEE 802.11ax for a single 20 MHz subchannel. The allocation index uniquely identifies each predefined RU assignment; for a given index, the remaining columns specify how the subchannel is subdivided into RUs and, consequently, how many STAs can be served simultaneously. For example, allocation index 0  partitions the 20 MHz subchannel into nine 26-tone RUs, whereas allocation index 1 partitions it into seven 26-tone RUs and one 52-tone RU, thereby reducing spectral granularity for one user in exchange for a wider RU and potentially higher per-user throughput. Indices expressed as ranges, such as 16–23, denote families of valid assignments that depend on the number of simultaneously scheduled stations, $n_{s}$, while the symbol “−” marks unassigned portions of the subchannel. In practice, the access point selects an allocation index that matches the scheduled set of STAs and their RU size requirements, which provides a standard-compliant mechanism to trade spectral granularity against per-user bandwidth within each OFDMA transmission opportunity.

### 2.2. TUA Mechanism

The IEEE 802.11ax amendment introduced the Trigger Uplink Access (TUA) mechanism, which plays a crucial role in facilitating coordinated uplink transmissions. Specifically, the 802.11ax standard defines the AP Trigger Frame as a control frame used to initiate TUA channel access for stations (STAs). This frame is broadcast by the Access Point (AP) and contains both common and user-specific fields to coordinate uplink transmission, particularly for Multi-User MIMO (MU-MIMO) operations [18].

The Trigger Frame in 802.11ax provides essential information in its common fields, which include the expected response frame length, bandwidth allocated for the response, and other control parameters required for the transmission process. These fields ensure that the AP and STAs are synchronised with the transmission parameters. In addition to the common fields, user-specific fields provide details about each device involved in the upcoming uplink transmission [27]. These fields typically include the following:

- Association ID: uniquely identifies the STA in the network.
- Uplink MCS (Modulation and Coding Scheme): indicates the modulation and coding rate that the STA should use for the transmission.
- Number of Spatial Streams: specifies the number of spatial streams allocated for MU MIMO.
- Target RSSI (Received Signal Strength Indicator): indicates the desired signal strength for the transmission.

TUA is the exclusive mechanism for initiating uplink MU transmissions in Wi-Fi, and its role in enabling efficient multi-user access is critical. However, because 802.11ax operates in a distributed manner, the AP does not have direct knowledge of the buffer status or data load of individual stations at any time. This lack of direct insight into the STA backlog presents a challenge for optimising the scheduling of MU MIMO and Orthogonal Frequency Division Multiple Access (OFDMA) transmissions. The effectiveness of these multi-user transmissions relies heavily on the coordination among stations, specifically their ability to report their uplink buffer status to the AP.

By coordinating the uplink buffer status across all participating stations, the AP can more effectively select the stations to engage in simultaneous MU-MIMO and OFDMA transmissions. This coordination enhances the efficiency of channel usage and ensures that the uplink transmission process remains synchronised, maximising throughput and minimising delays.

**Figure 3:**
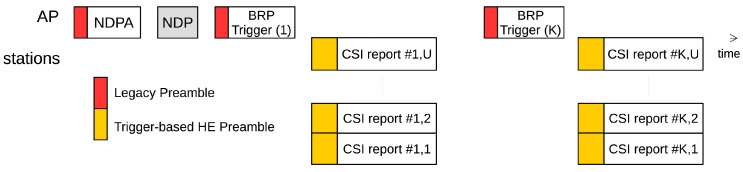
Data exchange in a trigger-based multi-user transmission in IEEE 802.11ax [18].

Figure 3 illustrates the procedure for acquiring Channel State Information (CSI) in Wi-Fi 6 through the Trigger-based Uplink Access (TUA) mechanism. In the time sequence, the AP first transmits a Null Data Packet Announcement (NDPA), followed by a Null Data Packet (NDP), which allows the stations to estimate the channel characteristics. The AP then sends a series of Beamforming Report Poll Trigger (BRP Trigger) frames, identified as BRP Trigger (1) up to BRP Trigger (K), requesting different groups of stations to report their channel state information. Each station responds with one or more CSI reports in the uplink direction, using High Efficiency Trigger-based Preambles (shown in yellow), while the AP’s initial messages employ Legacy Preambles (shown in red) for backward compatibility with earlier standards. Overall, the figure depicts how the AP sequentially gathers channel information from multiple stations, enabling more efficient multi-user transmissions through OFDMA.

### 2.3. TWT Mechanism

Target Wake Time (TWT) was first introduced in the IEEE 802.11ah amendment to support low power consumption in wireless networks, particularly for stations (STAs) with low traffic loads and periodic data transmissions. This mode allows the access point (AP) to control the wake-up time of STAs, thus saving energy by enabling STAs to remain in a low-power doze state when no data are being transmitted. The detailed operation of the TWT was initially defined in the 802.11ah standard and later extended in the IEEE 802.11ax amendment, which introduced additional features, such as a broadcast agreement and support for multi-user operation [28]. The TWT operates through two types of agreements: individual and broadcast. In the individual TWT, each STA must individually negotiate with the AP to set the parameters for the TWT operation. This negotiation occurs through management frames, such as beacon frames or association request/response frames, in which TWT-related elements are included as part of the standards. The TWT parameters in this case typically include the following:

- Target Wake Time: the specific time when the STA is scheduled to wake up and listen for incoming data.
- Wake Duration: the period during which the STA remains awake to receive data.
- Wake Interval: the time period between consecutive wake-up events (only applicable for periodic operation). Once the negotiation is complete, the STA switches to a doze state and remains inactive until a specified target wake time is reached.

In contrast, the broadcast TWT simplifies the process by allowing the AP to broadcast TWT parameters to all STAs via beacon frames. When an STA receives a beacon frame, it uses the TWT information to determine when to wake up and begin communication. The STA then enters the doze state again until the next target wake time, as specified in the broadcasted beacon frames. This method is more efficient in cases where multiple STAs need to synchronise their wake-up times without individual negotiations [28].

**Figure 4:**
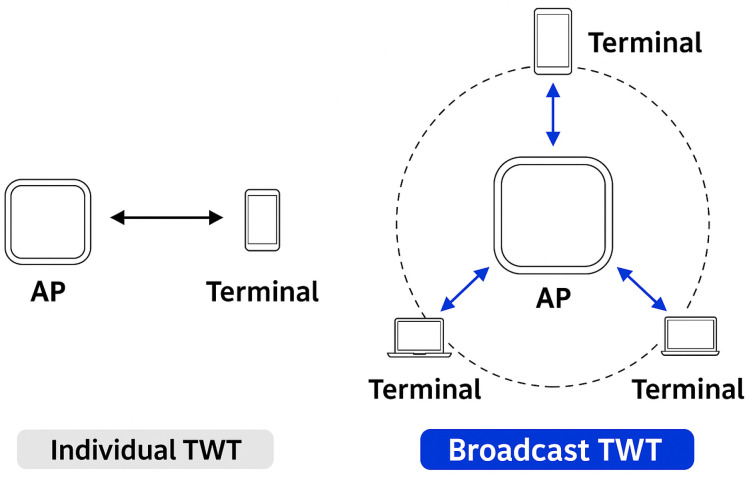
Operational modes of the TWT mechanism: individual and broadcast-based coordination.

The two modes of TWT operation, individual and broadcast, are conceptually illustrated in Figure 4. On the left, the individual TWT shows one-to-one coordination between the AP and each terminal. On the right, the broadcast TWT demonstrates how a single message from the AP can coordinate multiple terminals simultaneously.

## 3. Interference Models in Wi-Fi Networks

Interference is a critical factor influencing the performance of wireless local area networks (WLANs), particularly in unlicensed bands such as 2.4 and 5 GHz, where multiple technologies coexist. In the context of IEEE 802.11ax, which introduces OFDMA as a key mechanism for improving spectral efficiency and determinism, understanding and characterising the behaviour of the protocol under interference are essential. Unlike controlled simulations, real-world deployments are exposed to various interference sources that may either follow the Wi-Fi protocol or originate from foreign non-Wi-Fi devices. To evaluate the robustness of trigger-based MU-OFDMA uplink transmission under interference and compare it with conventional SU uplink transmission, this study defines two distinct interference conditions. The first involves Wi-Fi-based interference, which is used to analyse the impact of valid 802.11 frames on medium access. The second consists of Non-Wi-Fi interference, generated as RF noise within the 2.4 GHz band, to assess performance degradation in the presence of unstructured, protocol independent interference.

### 3.1. Wi-Fi-Based Interference

A realistic form of interference arises from Overlapping Basic Service Sets (OBSS), where stations receive valid IEEE 802.11 frames from neighbouring networks. These types of frames trigger physical and virtual carrier sensing, extend medium access delays, and increase contention. This is particularly relevant for dense deployments. Tardioli and Almeida [29] demonstrated that different commercial NICs interpret Wi-Fi interference heterogeneously, affecting MAC layer behaviours such as deferral and buffering. These effects complicate the performance guarantees of multi-AP environments. In Wi-Fi 6, BSS colouring offers mitigation strategies, but their effectiveness depends on the signal detection thresholds and AP coordination. Therefore, evaluating OFDMA under Wi-Fi interference is critical for quantifying its ability to efficiently recover from deferred access and quickly schedule multi-user transmissions once the channel is acquired.

### 3.2. Non-Wi-Fi Interference

In addition to structured contention, WLANs are exposed to unstructured interference from non-802.11 sources. Devices such as Bluetooth, ZigBee, microwave ovens, and industrial emitters generate noise that cannot be decoded by Wi-Fi receivers. These signals often evade virtual carrier sensing, causing packet corruption instead of orderly deferral. Bosch et al. [30] and Vikulov et al. [31] emphasise that Wi-Fi’s performance degrades significantly under such non-decodable sources. Unlike conventional full-band operation, OFDMA enables per-RU scheduling and can tolerate narrowband interference by reallocating traffic to unaffected subcarriers. Vikulov et al. [31] provide a model for partial RU allocation that shows measurable gains under adjacent channel interference, a condition analogous to narrowband jamming.

**Figure 5:**
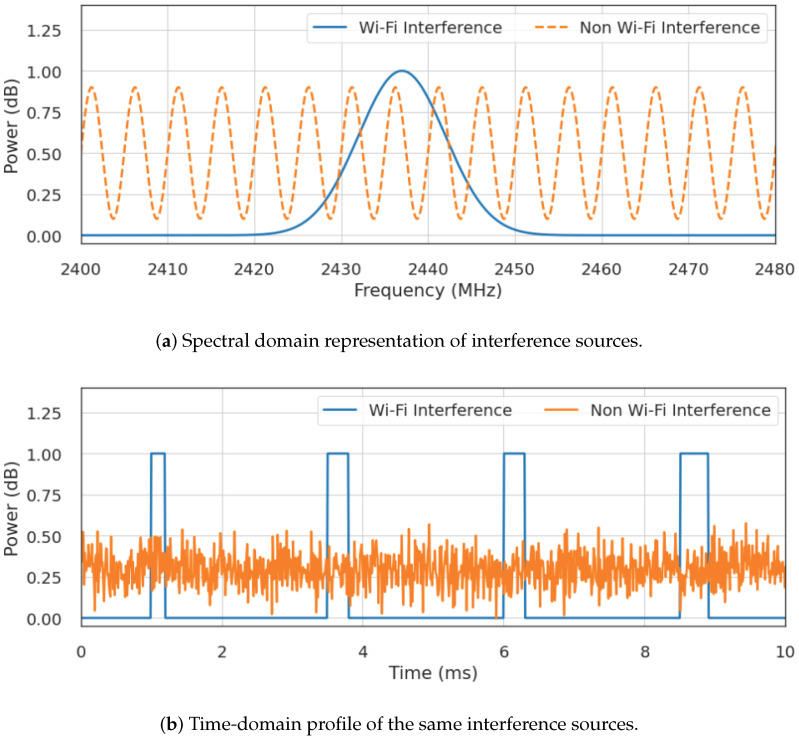
Characterisation of the two interference conditions.

The two interference conditions evaluated in this study are shown in Figure 5 and described below. Figure 5a presents the spectral domain representation, where the Wi-Fi interference appears as a concentrated, high-power signal centred at 2437 MHz, resembling a valid 802.11 transmission. In contrast, the non-Wi-Fi interference spans the full 2.4 GHz band with a repetitive and unstructured pattern, mimicking the behaviour of cross-technology RF sources, such as Bluetooth or industrial noise. Complementarily, Figure 5b depicts the time-domain profiles of both signals. Wi-Fi interference is characterised by discrete, periodic bursts that correspond to protocol-compliant frame transmissions, whereas non-Wi-Fi interference manifests as a continuous, low-level noise floor with no defined structure. Together, these figures demonstrate the contrasting nature of structured and unstructured interference and motivate the use of both types to assess the robustness of OFDMA under diverse real-world conditions. The use of both interference-type Wi-Fi frames and non-Wi-Fi as noise provided a comprehensive evaluation of OFDMA performance. The first type stresses MAC layer access and coordination, whereas the second tests physical layer resilience and spectrum flexibility.

## 4. Performance Evaluation

This section presents the experimental methodology designed to evaluate the proposed deterministic Wi-Fi network in a real testbed, complemented by numerical results presented elsewhere in the paper. For clarity, the compared transmission modes are denoted as conventional single-user (SU) uplink transmission and trigger-based multi-user OFDMA (MU-OFDMA) uplink transmission. In both configurations, OFDM is used as the underlying physical-layer modulation, whereas OFDMA refers to the multiple-access mechanism used in the multi-user case to allocate Resource Units within the available time-frequency resources among users. The implementation relies on Texas Instruments CC3301 and Nordic Semiconductor nRF7002DK development boards, together with an Asus AX6000 access point running OpenWRT, which enables integration with external applications for advanced network configuration.

To support this evaluation, we integrated additional firmware and software components on OpenWRT, the TI CC3301 platform, and the Nordic nRF7002DK platform in order to expose a static scheduling mode that can be configured directly from the AP operating system. This serves as a baseline test of deterministic operation, where scheduling decisions are fixed so that the same medium access schedule is enforced throughout the experiment, enabling controlled and reproducible timing behaviour.

In this baseline configuration, deterministic behaviour emerges from the combined action of Target Wake Time (TWT), Trigger-based Uplink Access (TUA), and Orthogonal Frequency Division Multiple Access (OFDMA). TWT establishes a periodic service interval for the station set under test and imposes a stable time structure for medium access. In the performance evaluation, the TWT schedule is periodic with a fixed service interval denoted by SI, which defines the TWT frequency as $f_{TWT}=1/SI$. Within each service interval, the AP uses TUA to initiate uplink transmissions at prescribed instants, avoiding contention and reducing timing uncertainty. In the baseline configuration considered in this work, the trigger period is set equal to the TWT service interval, i.e., $T_{trig}=SI=10$ ms, so that one scheduled trigger-based uplink opportunity is provided per service interval. The trigger frames embed the OFDMA resource unit assignment so that stations transmit concurrently using a fixed and repeatable frequency partition of the channel. Fixing the service interval, the trigger cadence, and the resource unit map minimises run to run variation and provides a clear first step validation of deterministic multi user operation on COTS platforms.

Concretely, the firmware and software modifications enable a manual schedule mode in which rate adaptation and dynamic resource-unit or station regrouping are disabled for the scheduled traffic class. The TWT configuration is set once at experiment start and kept constant, and the AP reuses a fixed trigger template that includes timing, the resource-unit map, and the relevant PHY constraints across all service intervals. The resulting operation is summarised in Algorithm 1. The initial steps define a fixed RU allocation map R together with PHY constraints P, followed by the one-time establishment of the TWT configuration for the station set. The periodic loop then mirrors the service-interval structure imposed by TWT: stations wake synchronously at the beginning of each SI, the AP issues trigger frames at a constant cadence $T_{trig}$, and OFDMA enforces the predetermined RU mapping for concurrent uplink transmissions. This simplified control flow makes explicit where temporal and spectral scheduling decisions are frozen in our experiments and clarifies how TWT, TUA, and OFDMA jointly realise repeatable and bounded uplink timing.
**Algorithm 1** Static interaction of TWT, TUA and OFDMA**Input:** Station set S, service interval SI, trigger period $T_{trig}$**Output:** Fixed uplink schedule in each service interval  1:Install fixed RU map R and PHY constraints P  2:Negotiate TWT agreements for S (once at experiment start)  3:**while** experiment is running **do**  4:     **TWT:** stations in S wake for the service period  5:     **for** k←1 to $\left\lfloor SI/T_{trig}\right\rfloor$ **do**  6:         **TUA:** AP transmits Trigger Frame at time $t_{k}$  7:         **OFDMA:** trigger carries R; each STA transmits in its RU using P  8:     **end for**  9:     **TWT:** stations return to doze until the next SI10:**end while**

### 4.1. Experimental Scenario

**Figure 6:**
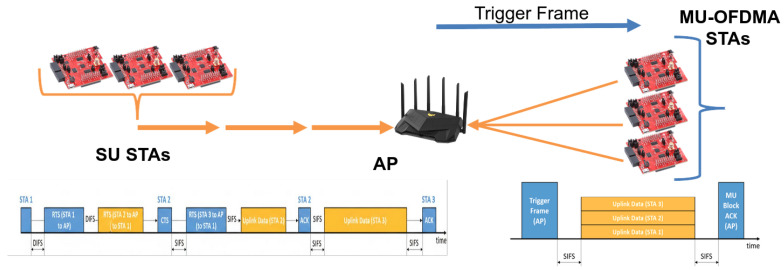
Conceptual architecture of the experimental deployment and control plane.

The testbed experiments were conducted in a controlled laboratory environment that was specifically configured to minimise external interference. The setup comprised a Wi-Fi 6 access point and three client stations, all equipped with IEEE 802.11ax-compliant interfaces. As noted above, Figure 6 provides a conceptual view of the system architecture. Initially, baseline performance measurements were conducted under conditions of minimal external interference to characterise the temporal and throughput performance of trigger-based MU-OFDMA uplink transmission versus conventional SU uplink transmission under a fixed, reproducible deployment. The objective was to quantify how each operational mode manages the available channel resources under controlled conditions, before introducing additional stress factors such as controlled interference and multi-client load.

To make this comparison explicit, in this paper, the reference configuration without OFDMA denotes an IEEE 802.11ax setup in which uplink transmissions follow conventional SU operation, whereas the scheduled configuration denotes trigger-based MU-OFDMA uplink transmission. Although OFDMA is a core capability of 802.11ax, commercial access points do not necessarily apply it continuously: it can be disabled by configuration, enabled only for selected traffic or stations, or replaced by conventional SU operation when the scheduler does not issue trigger-based allocations. Consequently, the SU versus trigger-based MU-OFDMA comparison adopted in this study provides a controlled baseline that isolates the impact of deterministic multi-user scheduling from the other PHY/MAC enhancements introduced by Wi-Fi 6.

Each scenario was executed repeatedly for 10 min and repeated 15 times, discarding the first 2 min of each run as a warm-up interval. This warm-up period accounts for transient effects at the beginning of each repetition, including association and buffering dynamics, rate adaptation stabilisation, and the convergence of the external control loop applying TWT and TUA-related settings. For each metric, we report the mean and standard deviation across runs, together with the corresponding 95% confidence interval (CI) computed across repetitions as $\bar{x}\pm t_{0.975,\;n-1}\;s/\sqrt{n}$. The CI is included to assess the robustness of the sample estimates and to verify that the observed differences are consistent with the measured run-to-run variability. Table 2 summarises the run parameters together with the key chipset, driver, firmware, and configuration parameters of the access point and the STA platforms.

The presets summarised in Table 2 are fixed to ensure reproducibility and to isolate the effect of deterministic scheduling. In particular, the TWT service interval and the trigger period are both set to 10 ms. This value was selected to maintain greater continuity of the scheduled operation and because 10 ms is the minimum value reliably supported by the system in our implementation. For the multi-client MU-OFDMA experiments, the RU allocation was fixed per scenario; specifically, the three-STA case used allocation index 16, corresponding to RU sizes of 106, 52, and 52 tones, while the one- and two-STA cases followed the corresponding subsets of the same static allocation framework. These timing and RU presets must be interpreted together with the selected channel bandwidth, since the bandwidth determines the pool of available RUs and therefore constrains the scheduling choices that can be exercised experimentally.

From this perspective, the experiments were intentionally conducted on a 20 MHz channel. In the targeted industrial setting, maximising peak bandwidth by bonding wider channels is not the primary objective; rather, the emphasis is on using narrower channels that are easier to protect from interference, simpler to prioritise, and more stable over time. For this reason, the combination of a 20 MHz channel with the fixed scheduling presets described above is sufficient to evaluate the deterministic behaviour of interest here. This does not imply that wider channel bandwidths are irrelevant. On the contrary, the RU structure summarised earlier shows the scalability potential of the same mechanisms: for example, the 26-tone RU configuration analysed in this paper would, in principle, scale from up to nine users in 20 MHz to up to seventy-four users in a 160 MHz channel. Accordingly, the present study should be interpreted as a focused validation of the low-level mechanisms on a practically relevant industrial channel width, while the standard-defined scaling to wider bandwidths remains structurally available once these mechanisms can be reliably controlled on COTS hardware.

To precisely quantify the comparative performance between trigger-based MU-OFDMA uplink transmission and conventional SU uplink transmission, a suite of widely recognised metrics from recent wireless communication studies was employed. These include:

- Throughput: Throughput is obtained from the receiver-side iperf3 statistics over the effective observation interval $T_{obs}$ after excluding the warm-up period. Unless otherwise stated, traffic is generated with the default iperf3 TCP configuration, in which the application uses the default write length reported by the tool (128 kB). Therefore, the experiments are expressed in terms of successfully delivered payload bits over time rather than in terms of a fixed application-layer packet size. For station *i*, the per-station throughput is defined as
  (1) $$Th_{i}=\frac{B_{i}}{T_{obs}},$$
  where $B_{i}$ is the number of payload bits successfully received from station *i* during $T_{obs}$. This per-station throughput $Th_{i}$ is the only throughput metric reported for conventional SU uplink transmission. For trigger-based MU-OFDMA uplink transmission, we additionally report the aggregate throughput, since multiple STAs may transmit simultaneously in different RUs. It is defined as
  (2) $$Th_{agg}=\sum_{i=1}^{N}Th_{i}=\frac{\sum_{i=1}^{N}B_{i}}{T_{obs}},$$
  where *N* is the number of simultaneously scheduled STAs. Hence, aggregate throughput is used only for MU-OFDMA cases, whereas SU cases are reported only through the per-station throughput $Th_{i}$.
- Latency and Jitter: Latency is evaluated from round-trip time (RTT) samples obtained with the standard ping utility during each experimental run. The reported latency corresponds to the average RTT over the valid samples collected within the observation interval and therefore captures the typical end-to-end response time perceived by the application. Jitter is used to quantify the temporal stability of that response and is computed from the dispersion of the RTT samples around their average value within the same run. In this way, low latency indicates fast network response, whereas low jitter indicates that such response remains temporally consistent from packet to packet. Both metrics are reported together because deterministic communication requires not only small delay, but also low variability in that delay over time.

#### Tests with Controlled Interference

Subsequently, the aforementioned tests were replicated with the deliberate introduction of two distinct types of controlled interference. This phase was designed to rigorously evaluate the resilience and adaptability of trigger-based MU-OFDMA uplink transmission compared with conventional SU uplink transmission under challenging spectral conditions.

- Wi-Fi Interference: This scenario involves generating additional traffic using common Wi-Fi 6 frames operating without OFDMA at a constant rate of 54 Mbps on the same channel. This condition emulates realistic coexistence scenarios prevalent in dense deployments, where diverse Wi-Fi configurations operate simultaneously. The primary aim is to evaluate the capacity of trigger-based MU-OFDMA uplink transmission for dynamic spectrum allocation and management under such interference and compare its performance against conventional SU uplink transmission.
- Non-Wi-Fi Interference: A continuous pulse of additive white Gaussian noise (AWGN) will be introduced, spanning the entirety of the 20 MHz channel in use as show in Figure 5. This simulates the wideband interference typically generated by non-Wi-Fi devices operating in the 2.4 GHz band, such as Bluetooth devices or industrial control systems. This test examined how both trigger-based MU-OFDMA and conventional SU configurations react to broad spectral perturbations, focusing on measuring effective throughput and latency.

Collectively, this experimental study provides a robust technical comparison between two IEEE 802.11ax operating modes: trigger-based MU-OFDMA uplink transmission and conventional SU uplink transmission. This comparison isolates the impact of RU-based multi-user scheduling and coordinated access on spectral efficiency and dynamic spectrum management, and it demonstrates the advantages of OFDMA under realistic propagation and interference conditions.

### 4.2. Evaluation of Resource Unit Allocation

To gain insight into the internal mechanisms of OFDMA transmission and its spectral characteristics, a set of theoretical experiments was carried out in MATLAB to model RU allocation as defined by the IEEE 802.11ax standard. A 20 MHz channel was subdivided into RUs according to the predefined allocation indices specified in the standard. Each allocation index maps to a unique partitioning of the frequency band, determining the number of RUs and, consequently, the number of users that can be scheduled simultaneously within a single transmission.

**Figure 7:**
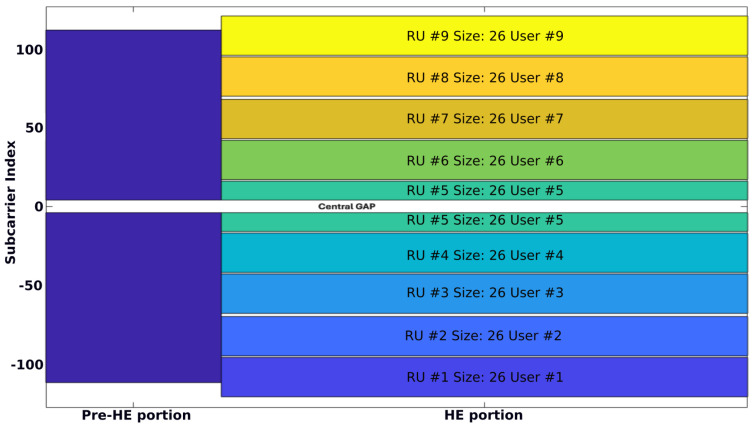
Subcarrier allocation for nine users under OFDMA.

The MATLAB WLAN Toolbox was used to examine how different RU configurations are mapped onto the total subcarrier allocation defined for a 20 MHz Wi-Fi 6 channel. For example, Figure 7 illustrates the RU assignment for a nine-user configuration. Each user is assigned a distinct RU, with nine users collectively allocated 26-tone blocks corresponding to allocation index 0, as shown in Table 1. The High Efficiency preamble (Pre-HE) is common to all users, whereas the HE portion is distributed across the allocated subcarriers.

This experiment was based on the complete 20 MHz channel bandwidth defined in Wi-Fi 6, comprising 256 subcarriers, out of which 242 are used for data while the remaining ones serve as pilot or null subcarriers. Notably, at the centre of the subcarrier index, specifically around subcarrier 0, a gap was observed. This division corresponds to a set of seven subcarriers that are deliberately left unused because the centre point of the spectrum contains a fixed component that carries no information. If that central component were used for data, it could introduce imbalance or unwanted shifts in the modulation. For that reason, those subcarriers are set aside and kept empty, preventing a static value at the spectral centre from affecting the signal’s linearity and overall quality.

**Figure 8:**
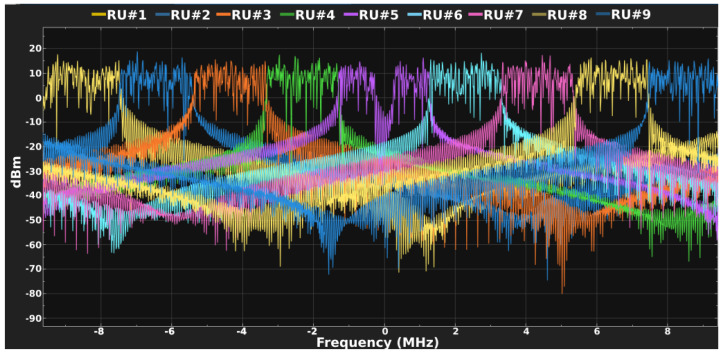
Orthogonal structure of the transmission in frequency domain.

For this configuration, Figure 8 displays the transmitted waveform in the frequency domain for the same nine-user configuration. Distinct spectral envelopes per user illustrate the non-overlapping, orthogonal nature of each RU, which ensures minimal intra-channel interference. The gap in the central frequency is the same as in the previous definition, is for control and management subcarriers.

**Figure 9:**
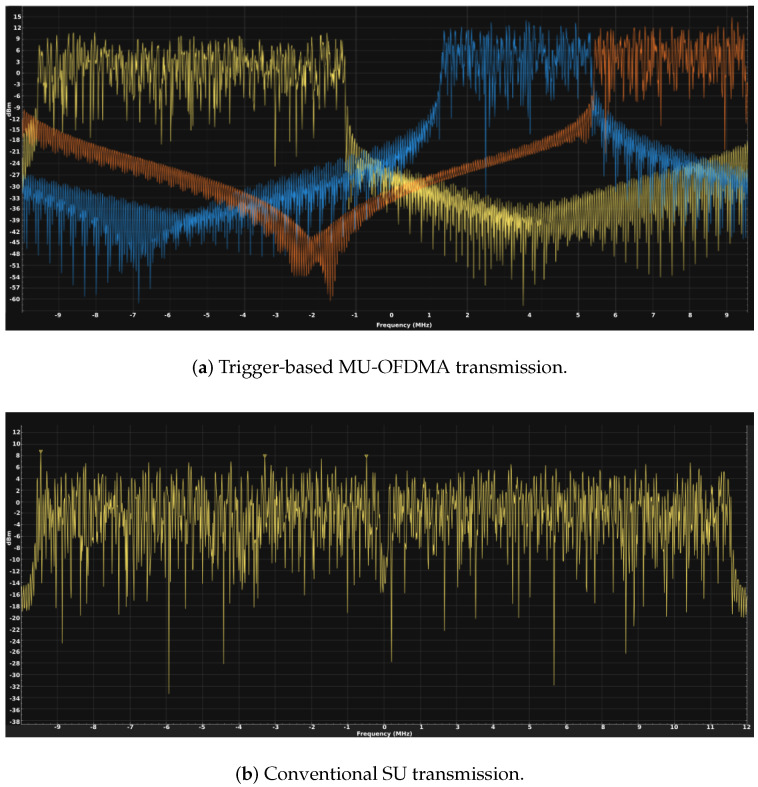
Spectral distribution comparison between trigger-based MU-OFDMA and conventional SU transmission.

The two waveforms shown in Figure 9 illustrate the fundamental difference in spectrum utilisation between trigger-based MU-OFDMA transmission and conventional SU transmission. In Figure 9a, the signal corresponds to an OFDMA configuration involving three users assigned RU sizes of 106, 52, and 52 tones, respectively, corresponding to allocation index 16. The spectrum is clearly divided into distinct, non-overlapping regions, each associated with a different user. This separation exemplifies the core principle of OFDMA: enabling simultaneous multi-user transmissions within the same channel bandwidth through orthogonal subcarrier allocation. In contrast, Figure 9b shows the waveform for conventional SU transmission. Although it uses the same 20 MHz channel, the entire spectrum is allocated to a single station. This exclusivity prevents other users from transmitting in parallel, leading to higher contention and reduced efficiency, especially in dense environments.

The comparison in Figure 9 underscores one of the key advantages of OFDMA: its ability to enhance spectral efficiency by enabling native multi-user access at the physical layer. Beyond the benefit of simultaneous transmissions, transmitting data over a smaller portion of the spectrum, as OFDMA does through the sub-division of Wi-Fi channels, has important physical-layer implications. Although each user operates over a narrower bandwidth, concentrating the same transmit power into a reduced spectral range results in a higher power spectral density. This leads to an improved signal-to-noise ratio (SNR), which can enhance the reliability and robustness of the system, particularly in noisy or interference-prone environments.

A practical illustration of this is found when comparing the spectral power envelope of the conventional SU uplink waveform in Figure 9b with that of the 106-tone RU in Figure 9a. This qualitative comparison is corroborated by our measurements. When the same total transmit power is concentrated into a 106-tone RU, the OFDMA signal exhibits approximately 3 dB higher power spectral density than the corresponding SU transmission that occupies the full 20 MHz channel. This behaviour follows directly from condensing the transmit power into a smaller spectral footprint, thereby increasing the energy per subcarrier. Consequently, OFDMA is particularly attractive for uplink scenarios, where client devices can benefit from improved robustness under constrained transmit power.

### 4.3. Results: Single-Client OFDMA Performance

**Figure 10:**
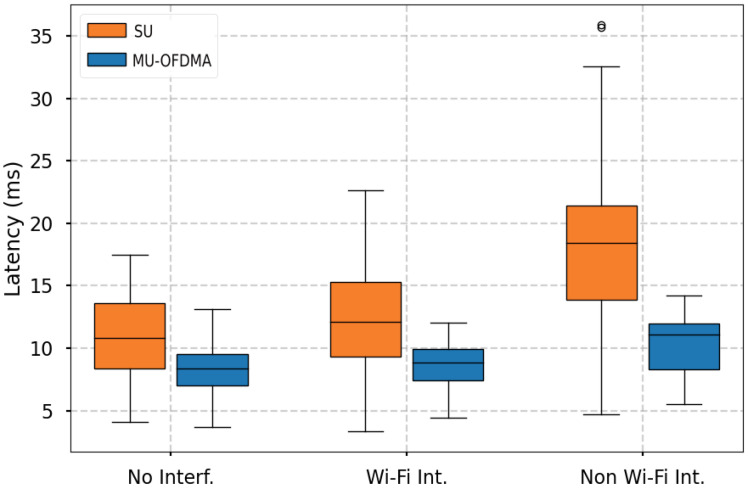
Latency comparison between trigger-based MU-OFDMA and conventional SU uplink transmission.

Figure 10 shows the latency behaviour of trigger-based MU-OFDMA uplink transmission compared with conventional SU uplink transmission under the interference conditions described in Figure 5. Under low or uncontrolled interference, MU-OFDMA consistently exhibited lower latency, with an average of 8.34 ms and a standard deviation of 1.90 ms, compared with 10.77 ms and σ=3.52 for SU transmission. This represents a 22.52% reduction, and the lower dispersion further indicates greater temporal stability even in the absence of interference. Under Wi-Fi interference, MU-OFDMA maintained this advantage in both mean latency and variability. It achieved an average of 8.71 ms with a standard deviation of 1.74 ms, while SU transmission reached 12.49 ms with a standard deviation of 4.17 ms, corresponding to a 30.24% improvement. The markedly higher variance observed in SU transmission highlights its susceptibility to pronounced fluctuations under intra-technology contention.

The behaviour under non-Wi-Fi interference further accentuates the performance gap between MU-OFDMA and conventional SU uplink transmission. MU-OFDMA achieved an average latency of 10.37 ms with a standard deviation of 2.19 ms, whereas SU transmission exhibited 18.70 ms and 6.66 ms, respectively, corresponding to a 44.54% latency reduction. This scenario also presents the largest difference in latency variability, with the higher dispersion observed in SU transmission reflecting its greater sensitivity to external interference and the associated contention and backoff processes. Consistently, the resulting 95% confidence intervals are [9.16,11.58] ms for MU-OFDMA and [15.01,22.39] ms for SU transmission, highlighting the substantially less stable latency behaviour under conventional SU uplink transmission.

This resilience of MU-OFDMA is primarily attributed to its use of narrow bandwidth, the improved signal-to-noise ratio it enables, and its scheduled medium-access approach combining TUA and TWT, which coordinates transmissions and eliminates network collisions. In contrast, conventional SU access based on CSMA/CA is considerably more vulnerable to high contention and random backoff, leading to severe latency degradation and significantly higher variability in congested environments.

**Figure 11:**
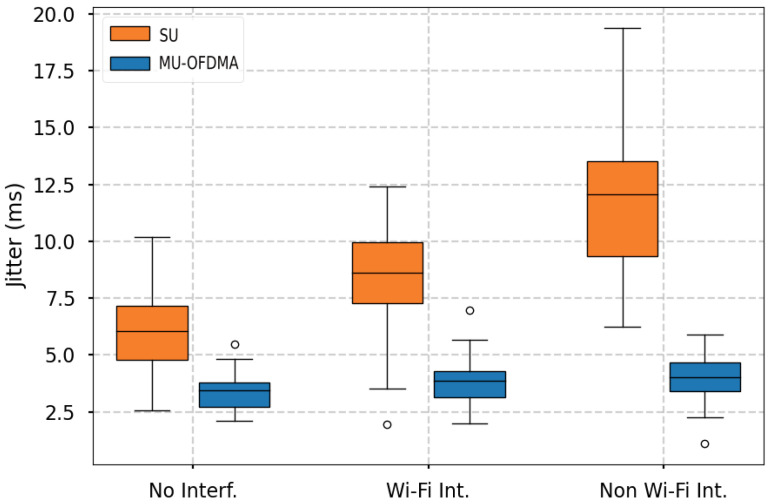
Jitter comparison showing temporal consistency improvements with trigger-based MU-OFDMA uplink transmission.

As depicted in Figure 11, the impact of interference on jitter is largely contained in MU-OFDMA transmissions, with only minimal increases observed. In contrast, conventional SU transmission is significantly affected. Beyond the average jitter, this becomes particularly evident when examining the standard deviation. The results show that MU-OFDMA maintains tightly bounded temporal variability across all scenarios, with σ between 0.71 and 0.95, indicating robust and repeatable behaviour under deterministic RU-based scheduling. Conversely, the standard deviation in SU transmission rises sharply with interference and load, increasing σ from 1.72 to 3.51. This pronounced growth highlights the vulnerability of contention-based access to timing fluctuations, whereas MU-OFDMA preserves the temporal stability required for delay-sensitive applications.

**Figure 12:**
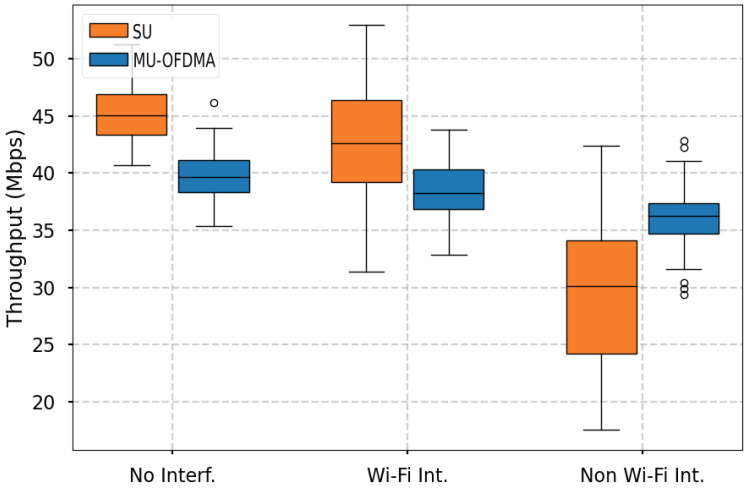
Throughput comparison between trigger-based MU-OFDMA and conventional SU uplink transmission.

The results in Figure 12 show a clear distinction in the throughput achieved by conventional SU uplink transmission and MU-OFDMA across the different interference scenarios assessed. In the absence of interference, SU transmission reaches throughput values close to the hardware-imposed limit, owing to its use of the full channel bandwidth, whereas MU-OFDMA yields slightly lower values due to the additional overhead associated with employing a 242-tone RU.

However, when Wi-Fi or non-Wi-Fi interference is introduced, conventional SU uplink transmission exhibits a pronounced degradation in throughput and increased variability, reflecting its full exposure to the spectrum and the consequent reduction in global MCS when any portion of the channel is impaired. In contrast, MU-OFDMA maintains a far more stable throughput, with only minimal reductions even under interference conditions. This stability stems from the segmented nature of MU-OFDMA transmissions, which limits vulnerability to interfered regions of the channel and reduces contention during medium access. Overall, the findings demonstrate that although SU transmission maximises throughput under stable conditions, MU-OFDMA provides superior robustness and consistency in the presence of real-world interference.

## 5. Multi-Client Performance Evaluation

To extend the single-client evaluation, additional experiments were conducted in a multi-client scenario involving three Wi-Fi 6 stations transmitting concurrently towards a common access point. The objective was to validate the capability of OFDMA to preserve deterministic behaviour when several clients contend for medium access.

The testbed configuration followed the same methodology as in Section 6, with the AP configured alternatively in two modes: (i) trigger-based MU-OFDMA uplink transmission, where Resource Units (RUs) were allocated per station, and (ii) conventional SU uplink transmission, where the entire channel was sequentially assigned to each user. All clients generated saturated traffic in uplink to ensure consistent medium contention. As in the single-client experiments, results are reported as averages and standard deviations computed across repeated runs under identical settings.

**Figure 13:**
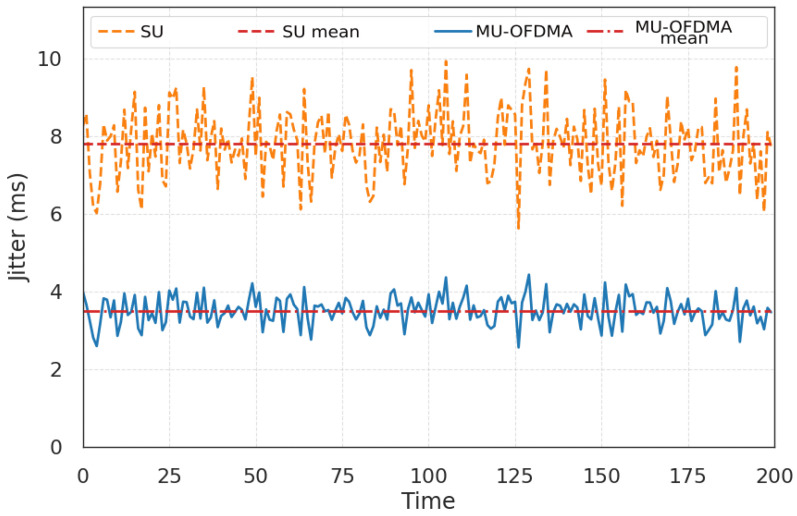
Temporal evolution of jitter for multi-client operation.

Temporal variability of the packet inter-arrival time, represented as jitter, is depicted in Figure 13. Trigger-based MU-OFDMA uplink transmission achieved a mean jitter of 3.51 ms, representing a 55.10% reduction compared with the 7.82 ms recorded for conventional SU uplink transmission. MU-OFDMA also demonstrated superior stability, evidenced by a markedly lower standard deviation of 0.68, compared with 1.61 for SU transmission. Under MU-OFDMA, jitter consistently remained below 5 ms with limited temporal fluctuation, whereas SU transmission exhibited a higher mean jitter and visibly larger variability, reflecting the inherent instability of contention-based channel access when supporting multiple active users. This degradation is primarily caused by retransmissions and randomised channel backoff delays, mechanisms which are effectively mitigated by the scheduled access inherent to the OFDMA process.

The comparative latency distribution was evaluated across one to three active clients. When trigger-based MU-OFDMA uplink transmission was used, average latency remained consistently low, ranging from 8.39 to 8.68 ms, with very small standard deviations of 1.97–2.01 ms, reflecting highly stable and predictable performance even under simultaneous transmissions. In contrast, conventional SU uplink transmission showed substantial increases in both average latency and variability, rising from 10.49 ms (σ = 3.03 ms) with a single client to 19.86 ms (σ = 5.08 ms) with three clients. These results correspond to relative latency reductions of 17.2%, 45.3%, and 57.6% for one, two, and three STAs, respectively, under MU-OFDMA operation. The low variability observed in MU-OFDMA demonstrates its ability to provide deterministic access times, while the larger standard deviations in SU transmission highlight the unpredictability caused by contention and backoff, emphasizing the superior stability and scalability of MU-OFDMA under multi-client conditions.

**Figure 14:**
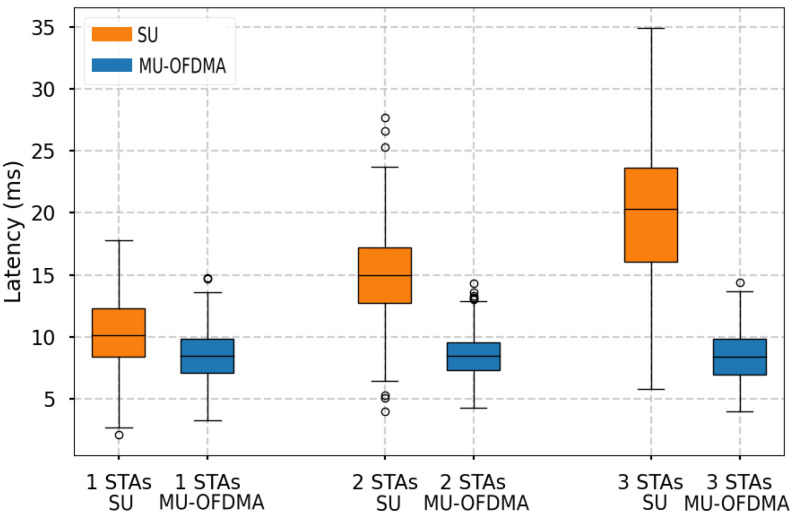
Latency distribution as a function of the number of active clients.

Furthermore, Figure 14 shows the same trend from a distributional perspective: MU-OFDMA keeps a narrow and nearly unchanged latency profile as the number of active clients increases, whereas SU transmission exhibits a progressive shift towards higher delay and wider dispersion. This divergence is consistent with the access principles of both schemes. MU-OFDMA coordinates simultaneous transmissions through RU allocation, while SU transmission relies on contention (CSMA/CA), where carrier sensing, backoff, and retransmissions increasingly penalise delay as the number of contenders grows.

The quantitative comparison is summarised in Table 3. Across all client counts, MU-OFDMA maintains almost constant mean latency of 8.5–8.7 ms, low standard deviation of 1.9–2.1 ms, and stable P95 values of 11.8–12.0 ms. Here, P95 represents the 95th-percentile latency and characterises tail behaviour: it is the latency threshold below which 95% of packet delays fall, while only the slowest 5% exceed that value. This metric is particularly relevant for deterministic communication, as it captures worst-case tendencies that are not fully reflected by the mean alone. In contrast, conventional SU transmission degrades steadily with load: mean latency rises from 10.36 ms to 20.27 ms, variability increases from 2.96 ms to 4.85 ms, and P95 grows from 15.10 ms to 27.86 ms between one and three STAs. Accordingly, the relative mean-latency reduction achieved by MU-OFDMA increases with density, from about 18% for one STA to 42% for two STAs and 57% for three STAs.

Overall, Figure 14 and Table 3 indicate that the gap between trigger-based MU-OFDMA uplink transmission and conventional SU uplink transmission widens as the number of contending stations increases. This widening is reflected by the joint divergence in mean delay, dispersion, and P95. The observed pattern is consistent with the underlying access mechanisms: contention-based SU access accumulates backoff and retransmission delays as load increases, whereas MU-OFDMA coordinates simultaneous RU-based transmissions and preserves bounded latency with lower variability. Therefore, although the experimental validation covers only up to three STAs, the consistency of the observed trends supports extrapolation, indicating that the same qualitative behaviour is likely to persist up to the nine-STA limit associated with the 26-tone RU configuration in a 20 MHz channel.

**Figure 15:**
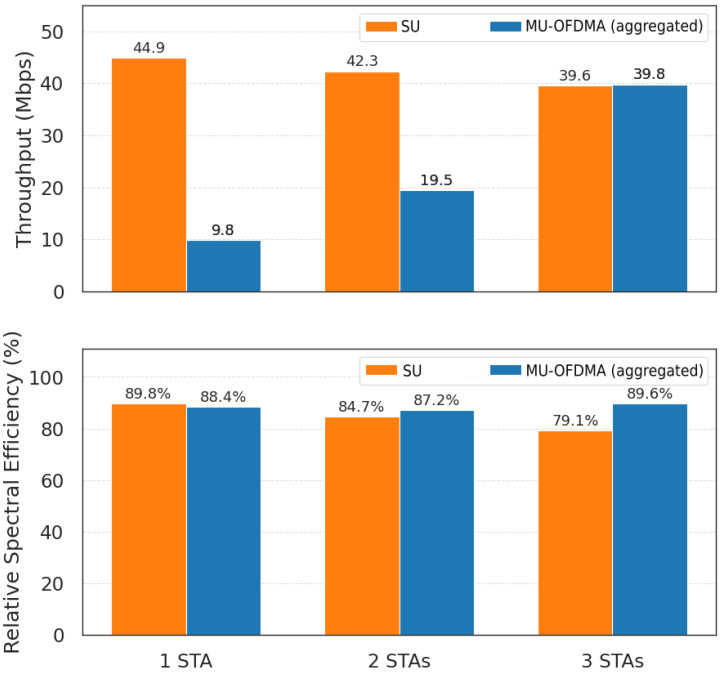
Throughput as a function of the number of active clients.

Figure 15 presents the aggregated throughput and corresponding spectral efficiency for the SU and MU-OFDMA cases under scenarios with one, two, and three simultaneous stations. In the MU-OFDMA case, each STA is assigned a distinct RU within the 20 MHz channel: the first two clients are allocated 52 tone RUs, while the third client receives a larger 106 tone RU. The total available channel capacity (50 Mbps) is therefore partitioned proportionally across these RUs, and the aggregated system throughput is computed as the sum of the individual RU throughputs.

Using this RU-based subdivision, the measured aggregated MU-OFDMA throughput increases from 9.8 Mbps with one STA, to 19.5 Mbps with two STAs, and up to 39.8 Mbps with three STAs. Although the absolute throughput per STA decreases as more RUs are allocated, the relative spectral efficiency remains consistently high, ranging from 88.4% to 89.6%. This reflects the fact that each RU is modulated and coded independently, and its efficiency is evaluated with respect to its own share of the spectrum, rather than the 20 MHz channel as a whole. The result is a near-optimal utilisation of each sub-band, with minimal guard-band overhead and no contention-driven delays, owing to the deterministic frequency-domain scheduling of MU-OFDMA.

In contrast, conventional SU uplink transmission shows higher absolute throughput per STA, between 39.6 Mbps and 44.9 Mbps, because each transmission occupies the full 20 MHz channel. However, only one STA can transmit at a time, causing the channel to be used sequentially rather than concurrently. As the number of active clients increases, the effective spectral efficiency decreases, from 89.8% with one STA to 79.1% with three, due to the cumulative effect of CSMA/CA backoff periods and collision avoidance overheads. Thus, while conventional SU uplink transmission provides higher instantaneous throughput to a single user, its contention-based MAC operation renders the system progressively less efficient as more clients share the medium.

The experimental findings highlight the deterministic advantages of MU-OFDMA in multi-client environments, but they also reveal effects that are not captured by reporting latency and jitter reductions alone. First, RU-based scheduling allows simultaneous transmissions, thereby avoiding the sequential allocation bottlenecks inherent in SU channel access. More importantly, the measured temporal behaviour remains largely insensitive to the number of active clients once the stations are isolated through RUs, which indicates that the main practical benefit is not only parallelism, but load decoupling at the MAC level. Second, the concentration of transmission power into narrower RUs improves signal-to-noise ratios, which contributes to enhanced robustness, particularly in uplink scenarios. This effect is relevant because the OFDMA configuration reduces the nominal bandwidth assigned to each STA, yet the experiments show that the narrower allocation can simultaneously preserve high relative spectral efficiency and improve resilience under interference. Third, the coexistence of OFDMA with mechanisms such as TWT ensures temporal predictability by aligning client transmissions within pre-defined activity windows, but this behaviour only becomes reproducible when the trigger-based uplink opportunities and TWT timing are configured consistently. Thus, the non-trivial observation is that deterministic-oriented operation emerges from the joint timing of TWT, trigger frames, and RU allocation, rather than from any single IEEE 802.11ax feature in isolation.

These observations clarify the difference between theoretical capability and device-level behaviour. In analytical and simulation-based studies, the scheduler is often assumed to have direct access to traffic state, timing, and RU allocation decisions. In the COTS platform evaluated here, those decisions are mediated by driver interfaces, firmware behaviour, and the subset of parameters exposed by the AP. Consequently, the experiments identify which standard-defined mechanisms can actually be coordinated on commercial hardware and which effects become observable once this coordination is enforced: station-level temporal isolation, robustness from narrow-RU power concentration, and repeatable uplink access windows. Although this manuscript evaluates a 20 MHz configuration only, the main implementation complexity lies in the coordinated management of TWT, trigger-based access, and OFDMA scheduling rather than in the absolute channel bandwidth itself. From this perspective, extending the same control logic to other bandwidths is expected to be largely transparent at the architectural level, even if the quantitative performance impact must still be validated experimentally. This is especially relevant in industrial settings, where the objective is often not to maximise peak throughput but to support frequent, predictable, and low-volume transmissions. Under such conditions, operation with small RUs is typically more representative than wideband allocations, since many industrial flows involve limited payloads transmitted periodically and with strict timing requirements. Taken together, these aspects show that OFDMA can improve timing stability and resource control in practical time-sensitive scenarios, while broader validation across additional bandwidths remains part of future work.

## 6. Conclusions

This paper has focused on the integration, development, and testing of the new high-efficiency (HE) mechanisms introduced in the IEEE 802.11ax standard, aiming to achieve a deterministic wireless network suitable for industrial and time-critical applications. The approach was first validated theoretically through numerical analysis and subsequently implemented on commercial off-the-shelf (COTS) hardware, where low-level driver modifications were introduced to fully enable the standard’s capabilities for deterministic operation. This dual validation confirmed the practical feasibility of trigger-based MU-OFDMA deterministic communication over Wi-Fi 6 in real environments using COTS hardware.

Experimental results demonstrated that the proposed configuration leads to significant temporal improvements. The use of OFDMA, in conjunction with Trigger-based Uplink Access (TUA) and Target Wake Time (TWT), resulted in a reduction in jitter by up to 55% and a decrease in average latency of about 57% in multi-client scenarios, achieving mean round-trip times close to 12 ms with highly stable distributions. Beyond these expected improvements, the experiments reveal that deterministic behaviour on COTS IEEE 802.11ax hardware is implementation-dependent rather than an inherent consequence of enabling OFDMA. Repeatable timing was only obtained when TWT scheduling, trigger-based uplink opportunities, and fixed RU assignment were jointly constrained. Under this configuration, MU-OFDMA did not merely reduce average latency; it also contained jitter and tail-latency growth as the number of active stations increased, whereas SU operation exhibited cumulative contention-dependent degradation. This indicates that scheduled RU allocation provides practical temporal isolation between stations, exposing a device-level effect that is usually hidden in analytical or simulation-based studies assuming ideal scheduler control.

In addition to its temporal benefits, the system maintained up to 90% of baseline throughput under interference, compared with 65–70% for conventional SU uplink transmission, and exhibited SNR gains of approximately 3 dB due to the power concentration within each RU. These results confirm that coordinated OFDMA operation can simultaneously improve robustness and predictability without sacrificing overall link reliability.

The reported results correspond to a single-AP Wi-Fi 6 deployment on COTS hardware operating over a controlled 20 MHz channel in the 2.4 GHz band, a configuration that is representative of an industrial operating regime in which spectral containment, interference robustness, and predictable access are prioritised over peak bonded throughput. Within this context, the main design challenge is not the absolute channel width itself, but the joint orchestration of TWT scheduling, trigger-based uplink coordination, and RU assignment. Accordingly, migration to wider IEEE 802.11ax channels should be largely transparent at the control-architecture level, since the same coordination framework can be preserved while the standard mainly changes the number and granularity of schedulable RUs. In that sense, wider channels are not expected to alter the governing access logic, but rather to provide an additional degree of freedom for increasing network scalability through a larger RU budget and a higher number of simultaneously schedulable transmissions. This interpretation is also aligned with typical industrial traffic profiles, which are characterised by periodic, low-payload, time-constrained transmissions and therefore tend to benefit more from stable allocation of small RUs than from wideband operation. The 20 MHz configuration therefore provides a technically meaningful baseline for evaluating the coordination mechanisms that govern deterministic behaviour, while the quantitative effect of bandwidth scaling remains an open dimension for future experimental characterisation.

Future work identified two main research directions. The first concerns advanced scheduling and orchestration, exploring how theoretical models of traffic orchestration can be combined with low-level reconfiguration mechanisms inspired by software-defined networking (SDN). This approach could enhance network adaptability and reliability while preserving deterministic behaviour. The second direction relates to the evolution of forthcoming standards. Technologies associated with Wi-Fi 7 and the ongoing Wi-Fi 8 trajectory extend the same design logic explored in this work, including finer resource allocation, stronger traffic differentiation, and tighter temporal coordination at the MAC level. In this sense, the present results are not only relevant to IEEE 802.11ax, but also transferable to newer Wi-Fi generations in which deterministic-oriented operation will increasingly depend on the coordinated use of scheduling, trigger-based control, and time-aware access mechanisms. These evolutions reinforce the vision outlined in this study: that deterministic wireless communication, originally a challenge for Wi-Fi, is now becoming a tangible reality for industrial grade networks.

## Figures and Tables

**Table 1:** 20 MHz Subchannel Resource Unit (RU) Assignment.

Allocation Index	20 MHz Subchannel Resource Unit (RU) Assignment								
0	26	26	26	26	26	26	26	26	26 *^a^*
1	26	26	26	26	26	26	26	52	
2	26	26	26	26	26	52		26	26
3	26	26	26	26	26	52		52	
4	26	26	52		26	26	26	26	26
5	26	26	52		26	26	26	52	
6	26	26	52		26	52		26	26
7	26	26	52		26	52		52	
8	52		26	26	26	26	26	26	26
9	52		26	26	26	26	26	52	
10	52		26	26	26	52		26	26
11	52		26	26	26	52		52	
12	52		52		26	26	26	26	26
13	52		52		26	26	26	52	
14	52		52		26	52		26	26
15	52		52		26	52		52	
16–23 (15 + $n_{s}$)	52		52		-	106 (1–8 STA) *^b^*			
24–31 (23 + $n_{s}$)	106 (1–8 STA) *^b^*				-	52		52	
…	…								
192	242								

*^a^* Last 26-subchannel assignment. *^b^* Valid assignment for 1–8 simultaneous STAs. ^-^ Unassigned RU. $n_{s}$ Number of STAs.

**Table 2:** Run parameters and testbed configuration for reproducibility.

**Run parameters and reporting**	
Run duration per scenario	600 s
Warm-up interval discarded	120 s
Number of repetitions	15 runs per scenario
**Static scheduling presets**	
TWT service interval and trigger period	Fixed and equal in the baseline configuration: $SI=T_{trig}=10$ ms.
RU allocation map	Fixed per scenario; in the three-STA MU-OFDMA case, allocation index 16 was used, corresponding to RU sizes of 106, 52, and 52 tones. The one- and two-STA cases used the corresponding subsets of this static assignment.
**Testbed hardware and software**	
Access point	Asus AX6000; OpenWrt 24.10.5; hostapd version; bandwidth 20 MHz.
STA platform 1	TI CC3300, Linux MPU v6.6 paired with a BeagleBone Black; internal 2.4 GHz antenna.
STA platform 2	Nordic nRF7002DK, Zephyr 4.3.0 standalone; internal antenna configuration.
Controller host	Ubuntu 24.04.3 LTS.

**Table 3:** Latency summary by number of active clients for conventional SU and MU-OFDMA uplink transmission.

N. STAs	Mean		Standard Deviation		95% CI		P95	
	SU	MU-OFDMA	SU	MU-OFDMA	SU	MU-OFDMA	SU	MU-OFDMA
1	10.36	8.51	2.96	1.97	[9.94–10.77]	[8.24–8.79]	15.10	11.86
2	15.04	8.67	3.90	2.07	[14.49–15.58]	[8.38–8.95]	21.44	11.96
3	20.27	8.73	4.85	1.90	[19.59–20.94]	[8.46–8.99]	27.86	11.79

## Data Availability

The data supporting the findings of this study are available within the article. Further inquiries can be directed to the corresponding author.
